# Use of physiological constraints to identify quantitative design principles for gene expression in yeast adaptation to heat shock

**DOI:** 10.1186/1471-2105-7-184

**Published:** 2006-04-03

**Authors:** Ester Vilaprinyo, Rui Alves, Albert Sorribas

**Affiliations:** 1 Departament de Ciències Mèdiques Bàsiques, Universitat de Lleida, Montserrat Roig 2, 25008-Lleida, Spain

## Abstract

**Background:**

Understanding the relationship between gene expression changes, enzyme activity shifts, and the corresponding physiological adaptive response of organisms to environmental cues is crucial in explaining how cells cope with stress. For example, adaptation of yeast to heat shock involves a characteristic profile of changes to the expression levels of genes coding for enzymes of the glycolytic pathway and some of its branches. The experimental determination of changes in gene expression profiles provides a descriptive picture of the adaptive response to stress. However, it does not explain why a particular profile is selected for any given response.

**Results:**

We used mathematical models and analysis of *in silico *gene expression profiles (GEPs) to understand how changes in gene expression correlate to an efficient response of yeast cells to heat shock. An exhaustive set of GEPs, matched with the corresponding set of enzyme activities, was simulated and analyzed. The effectiveness of each profile in the response to heat shock was evaluated according to relevant physiological and functional criteria. The small subset of GEPs that lead to effective physiological responses after heat shock was identified as the result of the tuning of several evolutionary criteria. The experimentally observed transcriptional changes in response to heat shock belong to this set and can be explained by quantitative design principles at the physiological level that ultimately constrain changes in gene expression.

**Conclusion:**

Our theoretical approach suggests a method for understanding the combined effect of changes in the expression of multiple genes on the activity of metabolic pathways, and consequently on the adaptation of cellular metabolism to heat shock. This method identifies quantitative design principles that facilitate understating the response of the cell to stress.

## Background

Cells mount adaptive responses that involve changes in gene expression in order to survive environmental stress. These changes lead to shifts in the activity of the corresponding proteins and often to observable phenotypes. In each stress condition, the effectiveness of the cellular responses is subordinated to a variety of functional constraints. Ultimately, any change in gene expression and protein activity must allow the cell to survive the stress and to maintain a metabolism that allows reproduction (or at least survival until stress no longer exists and normal reproduction can occur). Out of the wealth of physically possible gene expression changes, only a small set of patterns allows the cell to effectively survive a given type of stress. For instance, a complex and well-defined program of gene expression changes is activated when yeast is exposed to a mild heat shock caused by a temperature shift from 25°C to 37°C [1,2]. Changes in gene expression as a consequence of this environmental stress are mostly transient. The highest number of transcriptional changes is observed between 10 and 20 minutes after the shock. Even when heat shock is maintained, after this initial period the overall gene expression returns to a pattern almost identical to that under basal conditions [3-5]. These transient changes are necessary to shift the metabolism of the cell to a new steady state that is adapted to the stress conditions.

Metabolic states induced by different types of stress share common features. For example, heat shock also induces adaptation responses to other stresses [6]. Cells that are adapted to a mild heat shock become resistant to larger temperatures shifts [7], to oxidative stress [8], to osmotic stress and to freezing [9,10]. Physiological events that are common to the different types of stress responses include cell cycle arrest [11,12], changes in cell wall architecture [13,14], increase in the synthesis of heat shock proteins (most of them chaperones), and increase in the concentration of small protective molecules (glycerol and trehalose) [6,15,16]. Trehalose acts in synergy with chaperones in stress resistance. Moreover, trehalose preserves the structure of membranes, protects proteins against denaturation and aggregation [17], and is required for conformational repair of heat-damaged glycoproteins [18]. Trehalose also apparently protects DNA and lipids [19].

An adjustment of cellular energy metabolism is also required in response to stress. A higher energy supply is needed, for example, to guarantee the functionality of chaperones and of the plasma membrane H^+^-ATPase [16]. This leads to an increase in the rate of oxygen respiration, which promotes up-regulation of the mechanisms for protection against oxidative damage [20]. There is also an increase in the production rate of reducing equivalents in the form of NADPH, as they are necessary to balance the redox state of the cell. This balance is challenged due to an increased utilization rate of NADPH within several redox cycles that protect cellular components against oxidation (for example the glutathione and thioredoxine cycles [21]). Additionally, NADPH is needed to produce new saturated lipids that reduce cell membrane fluidity [16,22].

**Figure 1:**
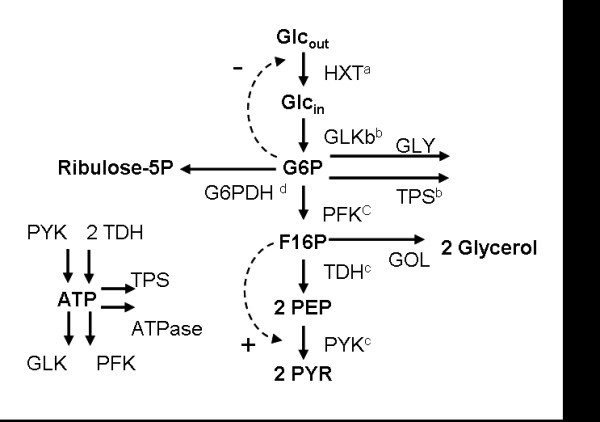
**Scheme of the modelled pathways and ranges used for generation of the *in silico *GEPs**. Our model includes the core of the glycolytic pathway and the first step of the pentose phosphate pathway, It also accounts for the synthesis of glycogen, trehalose and glycerol. Glcout: Extracellular Glucose; Glcin: Intracellular Glucose; G6P: Glucose-6-phosphate; F16P: Fructose-1,6-biphosphate; PEP: Phosphoenolpyruvate; PYR: Pyruvate; HXT: Hexose transporters (HXT1–4, HXT6–8, HXT12); GLK: Glucokinase/Hexokinase (GLK1, HXK1, HXK2); PFK: Phosphofructokinase (PFK1, PFK2); TDH: Glyceraldehyde-3-phosphate dehydrogenase (TDH1, TDH2, TDH3); PYK: Pyruvate kynase (PYK1, PYK2); GLY: Production glycogen; TPS: Trehalose 6-phosphate syntase complex (TPS1, TPS2, TPS3); G6PDH: Glucose 6-phosphate dehydrogenase (ZWF1). Range of fold changes expression explored in the simulations: (a) Gene allowed to increase its expression by up to ten times its basal level; 10 uniformly spaced samples. (b) Gene allowed increasing its expression by up to nineteen times its basal level; 13 uniformly spaced samples (c) Gene expression allowed to change between ¼ and four times the basal value; 7 samples taken, at levels of expression that are 4×, 3×, 2×, 1×, 1/2×, 1/3× and 1/4× that of its basal level. (d) Gene allowed increasing its expression by up to eight times its basal level; 8 uniformly spaced samples.

The core of the glycolytic pathway and its main branches capture most of the physiological changes previously described (Fig. 1). This is a relatively simple and well characterized metabolic system [23,28] that offers an ideal scenario to explore the yet unidentified underlying functional rules that shape the transcriptional changes. Furthermore, one should expect that biological insights obtained from the analysis of the response to a given type of stress may help in understanding the response to other types of stress. Evolving an adaptive response to environmental stress is a multiobjective optimization problem that involves metabolic changes at different levels such as fine-tuning of fluxes, of protein activities, of metabolite concentrations [29], and of the dynamic of the time responses [25]. Ultimately, the stress response is both constrained by the network structure of gene and protein interactions and by the need to assure the metabolic homeostasis responsible for the survival of the cell under different conditions. Consequently, adaptation to a given type of stress should not compromise the response to other types of stress.

Although the core metabolic response to different types of stress is similar, adaptation to each stress is achieved through different quantitative strategies. There is extensive overlap among the sets of genes that help the cell survive to different types of stress, although the quantitative changes in the expression for the common genes are not the same in each type of adaptive response [3-5]. This suggests diverse adaptive solutions to the various types of stress.

Due to the complexity involved in the cellular response to stress, understanding the physiological principles that explain the observed changes in enzyme/protein levels requires an integrated approach that focuses on the systemic behaviour of cellular pathways [30,32]. This analysis must be based on a sound understanding of the metabolism involved and it must be tested in front of experimental data. In this context, microarray data provide basic semi-quantitative information about gene expression changes that help characterizing the gene pattern corresponding to a given cellular response. For example, when yeast is exposed to heat shock the *gene expression profiles *(GEPs) from three different experimental databases [3,5] show high values of over-expression for the hexose transporters (HXT), the glucokinase/hexokinase (GLK) and the trehalose syntase complex (TPS). The glucose-6-phosphate dehydrogenase (G6PDH) and the glyceraldehydes-3-phosphate dehydrogenase (TDH) are also slightly over-expressed. On the other hand, phosphofructokinase (PFK) and pyruvate kinase (PYK) do not seem to be affected under heat shock conditions.

Although these experiments provide a general picture of the required gene expression changes, evaluation of the effects of these changes on the metabolic state of the cell is not straightforward. Therefore, understanding GEPs in terms of physiological changes requires a systemic approach. Mathematical models of the pathways involved in the physiological response provide a helpful tool for investigating this problem [27,28,32-36]. A critical point in this analysis is the appropriate evaluation of the cellular consequences resulting from gene expression shifts that are caused by a given environmental change. This composite analysis must always be put in the context of the existing biological knowledge about cellular physiology.

In this work we identify and test functional constraints that shape the naturally evolved physiological response of *Saccharomyces cerevisiae *to heat shock. First, we investigate the correspondence between perturbation of enzyme activities and physiological adaptive changes using a mathematical model. Then we show that the observed pattern of gene expression changes that characterizes the response of *S. cerevisiae *to heat shock can be explained by a defined set of functional principles. These include constraints on the changes in the fluxes and the concentration of metabolites, on the cost associated with the changes in gene expression, and on the synergistic effects between changes in different parts of the system. Our work underlines the importance of mathematical models in identifying relevant functionality criteria and evaluating their importance in molecular systems biology.

## Results

### A method for evaluating quantitative design principles for gene expression in yeast adaptation to heat shock

The observed GEPs in response to heat shock are a result of evolution and should correspond to design principles leading to an appropriate adaptive response. For example, understanding the increase in gene expression for the HXT is intuitive because glucose is required for an increase in ATP synthesis and other related changes. However, understanding why the experimentally observed change in gene expression is of about 6- to 8-fold [27,34] instead of some other value is not as intuitive. Moreover, while explaining this observation one must take into account the changes in the expression of genes that code for other enzymes. For example, GLK should compensate the increased uptake of glucose, avoiding an accumulation of this metabolite that could compromise viability of the cell. A systemic analysis of the metabolic pathways involved in the response is required to properly evaluate the possible changes in gene expression and their impact on physiological fitness.

Previous work has shown the optimality of an experimentally observed GEP when compared to a small set of alternative theoretical GEPs [27]. This analysis provided a preliminary insight into the metabolic changes that allow yeast to adapt to heat shock. Taking those results as a starting point, we suggest a more general modelling based approach to investigate this problem in further detail. In our *in silico *approach we use a mathematical model to exhaustively scan the gene expression space. We evaluate the effect of gene expression changes, according to a predefined set of functionality criteria that expand and generalize those of Voit & Radivoyevich [27]. We then identify, from several millions of alternative GEPs, the set that, according to the functionality criteria, lead to an appropriate physiological response. At this stage we validate the selected set of profiles by comparison with the data from available microarray experiments. This approach provides a more general insight into the underlying quantitative design principles that lead to the experimentally observed GEP. Our approach can be applied to analyze different stress responses. In this paper we focus on heat shock response in yeast as a benchmark problem for the approach because of the following:

i) A valid mathematical model that describes most of the relevant processes exists [27,37-39].

ii) Comparison with previous work is possible and can help in defining the set of functional criteria [27].

iii) There are experimentally determined GEPs from different microarray experiments performed under very similar heat shock conditions that help validate our predictions [3-5].

iv) The method can provide further insight into the biological response.

In detail, the suggested approach, applicable to other types of adaptive response and in other organisms, consists of the following steps:

1) **Build a mathematical model that includes the main metabolic pathways involved in the response to the considered environmental change. **These must be identified from the literature and from a gene ontology analysis of the available microarray data. The model will be used to investigate the effect of changes in gene expression (seen as changes in enzyme activity) on precise metabolic characteristics (fluxes, metabolite levels, cost of over-expression, etc.).

2) **Define physiological criteria that should be met after adaptive response to heat shock. **These criteria must be established based on available information on the physiology of the relevant problem. In the case of heat shock in yeast, there is abundant information in the literature that helps in defining such criteria. Ideally, one should be able to use experimental data in order to estimate the cut-off values for each criterion. This estimation should take into account both the observed experimental measurements and the biological and experimental variability. To make sure that results do not fundamentally change and are qualitatively robust we should also check how sensitive the final result is to this variability. If available information does not help in estimating cut-off values for some criteria, one can use internal standards of the *in silico *population of profiles under study (see next point). One way to implement these internal standards is by selecting a fraction of profiles that are the best performers according to the relevant criterion.

3) **Generate an exhaustive set of alternative GEPs *in silico *and test the metabolic changes induced by each profile. ***In silico *GEPs are generated by combining different values for each gene. Then, fold-changes in gene expression are translated into changes in enzyme activities. These activities are parameters in the model and will induce a new steady-state with the corresponding fluxes and metabolite levels. In our case, we have simulated more that 4 million GEPs. For each GEP, the systemic response of the model in terms of fluxes and concentrations is computed.

4) **Select those GEPs that fulfil the physiological criteria. **Clearly, not all the considered changes generated in 3) will produce the appropriate response, defined as in 2). For example, as we discussed in the Introduction, heat shock response increases ATP consumption. If the gene expression for glucose transporters is not increased appropriately, the cell may be compromised, as it will not produce enough ATP to match the increased consumption rate. But an increase in glucose leads to side effects that can compromise the overall response. Thus, as it happens in the actual response of yeast to heat shock, compensation for undesired effects would require additional modifications. The criteria defined in 2) constrain the possible changes by checking its effects at the physiological level. Overall, only GEPs that simultaneously match all physiological effectiveness criteria defined in 2) are selected.

5) **Analyze the characteristics of the selected GEPs. **Different pictures can emerge from this analysis. First, it may occur that the final set of predicted GEPs is tightly clustered in expression space and includes the experimental GEPs. This would suggest that most of the functional criteria that are important in defining design principles for an adaptive response to that stress have been identified, at least for the set of genes under consideration. A second possibility is that the predicted GEPs are randomly spread out through expression space or that, although they cluster in a well-defined region, the experimental GEPs are not included in that region. This would suggest that either the criteria that have been used for selection or the genes that are being considered are not important for the stress response. A third situation that may happen is that, with some exceptions, most of the genes being considered cluster in the predicted gene expression region and that these are well matched to the corresponding genes in the experimental GEPs. This would suggest that some physiological criteria for effectiveness of the response have yet to be identified. The set of genes whose changes are not clustered in expression space provides guidelines towards identifying new physiological criteria. Once these have been tentatively identified and points 2) through 5) have been repeated, the analysis will tell if the newly identified criteria are relevant. Finally, it may happen that groups of predicted GEPs cluster into discrete orthogonal regions of the gene expression space. Assume that the response to the relevant stress in closely related organisms has been studied and that the experimental GEPs for these responses do not overlap and are found in two or more of the clusters. This suggests a situation with different and equally well adapted types of response, where the selection between the different GEPs may be a consequence of the organism's life history throughout evolution. Whatever the case is, the analysis proposed here will help in understanding biological adaptation in stress response.

### Mathematical model and prediction of the physiological performance for a GEP

The theoretical analysis of biological systems using mathematical models led to the inference of general biological design rules for different classes of biological systems, including gene circuits [40-42], metabolic pathways [43-55], signal transduction networks [56], and whole genomes [33,34,57-60]. Mathematical models are especially useful in this type of analysis because they allow for an exhaustive and systematic analysis of responses given by alternative models to the same stimulus. This provides a tool for testing hypothesis about the underlying design of systems [25,28], as some specific designs will not exhibit certain types of behaviour. Recently, some of the hypothesis about design principles in biological circuits have been experimentally tested and confirmed [57,61-63]. This provides a strong support to the further development of the many research programs that use mathematical models to analyze biological design principles.

In order to appropriately analyze the effect of changes in enzyme activities on metabolism, a mathematical model that allows the calculation of changes on fluxes and concentrations is required. Ideally and for accuracy's sake, one would like to build a model using the exact non-linear mathematical functions that describes the mechanism of each individual enzyme process. However, often this mechanism is unknown. When it is known, in most cases there is not enough reliable information to estimate realistic parameter values. An option is to use functional formalisms that, applying theoretically well supported mathematical approximations allow the determination of parameter values from semi-quantitative estimations of concentrations and fluxes. Among other possibilities, there is a long tradition of using linear mathematical approximations (e.g. [64,65]). These are mathematically tractable but lose the non-linear character of the processes they seek to represent. As an alternative that overcomes these limitations, the power-law representation [34,44,66,73] is a formalism that is tractable at steady state while retaining some of the non-linearity of the processes themselves. This is of special interest in application to the analysis of biochemical systems. In our work we use a model build using the power-law formalism. This model considers glycolysis, the biosynthesis of glycerol, trehalose, and glycogen and the first step of the pentose phosphate pathway (Fig. 1). This is a well established minimal model [27,37,39,74] that has been previously used to explore adaptation to heat shock and to aid in the search for functional constraints on gene expression during adaptation [27]. Technical details on model definition and parameter values are given in the Methods section and in the literature [27,37,39].

In our analysis we assumed that any change in the expression of a gene leads to an equivalent change in the activity of the enzyme coded by the gene [27]. This is justified by the fact that response to heat shock is generally regulated at the level of transcription [75-78]. Additional evidence for this type of regulation comes from the strong correlation that is found between transcriptional changes, amount of protein [79,80] and mRNA stability [81]. This correlation is particularly strong for glycolytic enzymes [82-84]. TDH and PYK are exceptions to this one-to-one correspondence between gene expression changes and enzyme activity changes. Post-transcriptional activation of TDH [85] and PYK [86] occurs in heat shock response. This activation was taken into account by considering that TDH enzyme activity changed by 1.5-fold with respect to the changes in TDH gene expression. Similarly, PYK enzyme activity was considered to change 5-fold more than PYK gene expression as suggested by Voit & Radivoyevitch [27]. Using the same approach suggested by these authors, if a reaction was catalyzed by a single enzyme, the change in enzyme activity was matched to the change of gene expression measured by the microarray; if the reaction was catalyzed by several enzymes or isoenzymes, microarray values were weighted by the number of mRNA copies per cell of each isoform obtained from Genome-Wide Expression Page [87,88].

### Deleterious gene expression profiles

A systemic analysis of the *in silico *generated GEPs reveals 0.57% of cases in which no stable steady state exists after the change in gene expression. Most of these profiles result in low activity for the enzymes of glycolysis's first steps and in high activity for the enzymes of the lower part of the glycolytic pathway. A biological interpretation of why these cases would be deleterious can be as follows. This pattern of enzyme activities excessively depletes the concentration of intermediate metabolites and thus prevents an adequate heat shock response because not enough material flows through the pathway. An analysis of the *Saccharomyces Genome Database *(SGD) [89] shows that deletion mutants in the early enzymes of glycolysis have phenotypes that are almost always severe and usually lethal. Deletion mutants in the enzymes of the lower part of glycolysis have defective growth but are rarely lethal. The difference is especially noticeable under stress conditions (data not shown). This suggests that a decrease in the activity of these early enzymes is deleterious even under normal conditions, which supports our interpretation.

### Flux-based criteria for functional effectiveness that evaluate physiological adequacy of the response to heat shock

Since a given GEP results in changes at the metabolic level both in fluxes and in concentrations, we can evaluate the appropriateness of each pattern by defining functional criteria that correspond to observed metabolic changes after heat shock. Based on the analysis of the literature and on a preliminary analysis of the model, we considered the following functional criteria based on flux constraints to evaluate the performance of each GEP:

**C1. Changes in gene expression must allow for an increase in the rate of ATP production. **This is so because the response to heat shock increases the activity of proteins that use large amounts of ATP, such as chaperones and the plasma membrane H^+^-ATPase [16]. The rate of ATP production after heat shock has been measured to be about five times that of the basal state of the cell [90,92]. Because of biological variability and the noise typical of experiments, we have allowed for a 40% variability on this value and select GEPs that have an ATP production flux that is at least three times higher than that of the basal steady state.

**C2. The rate of trehalose synthesis must increase after heat shock. **As mentioned in the introduction, trehalose needs to be synthesized by the cell in response to heat shock in order to protect lipids, proteins and DNA [15,17]. The concentration of trehalose after heat shock has been experimentally determined to range from less than ten times to about one hundred times that of the pre-stress steady state in one hour [19,91-93]. At 37°C there is an increase in the net productive flux of trehalose of approximately forty times with respect to the pre-stress steady state [93]. We allow a 40% variability to this estimation and select as cut-off value a rate of trehalose production that is twenty five times higher than that of the basal steady state. If a GEP allows an increase in the flux of trehalose synthesis that is more than twenty five times that of the basal steady state, we select the GEP as producing enough trehalose.

**C3. Changes in gene expression must allow for an increase in the flux of NADPH production after heat shock. **As previously described, this increase is fundamental because more NADPH is needed for the biosynthesis and redox protection of cellular components. There are no experimentally determined values for the increase in this flux during heat shock. In oxidative stress, a 2.1-fold induction of NAD(P)H dehydrogenase is observed [94]. Because NADP response in oxidative stress is known to be similar to that in heat shock [94,98], we chose a cut-off value for the increase in the productive flux of NADPH of twice the basal steady state flux.

### Constraints on gene expression caused by flux-based criteria for functional effectiveness

Criteria C1-C3 consider the need for an increased synthesis of trehalose, ATP and NADPH. When the criterion C1 was used as a filter, 45.13% of all tested GEPs were selected. The criterion C2 selected 60.95% of all tested GEPs. Finally, 85.86% of all tested GEPs were selected by the criterion C3. Simultaneous application of all three functional criteria C1-C3 selected 1,290,454 (27.83%) GEPs (Table 2).

**Figure 2:**
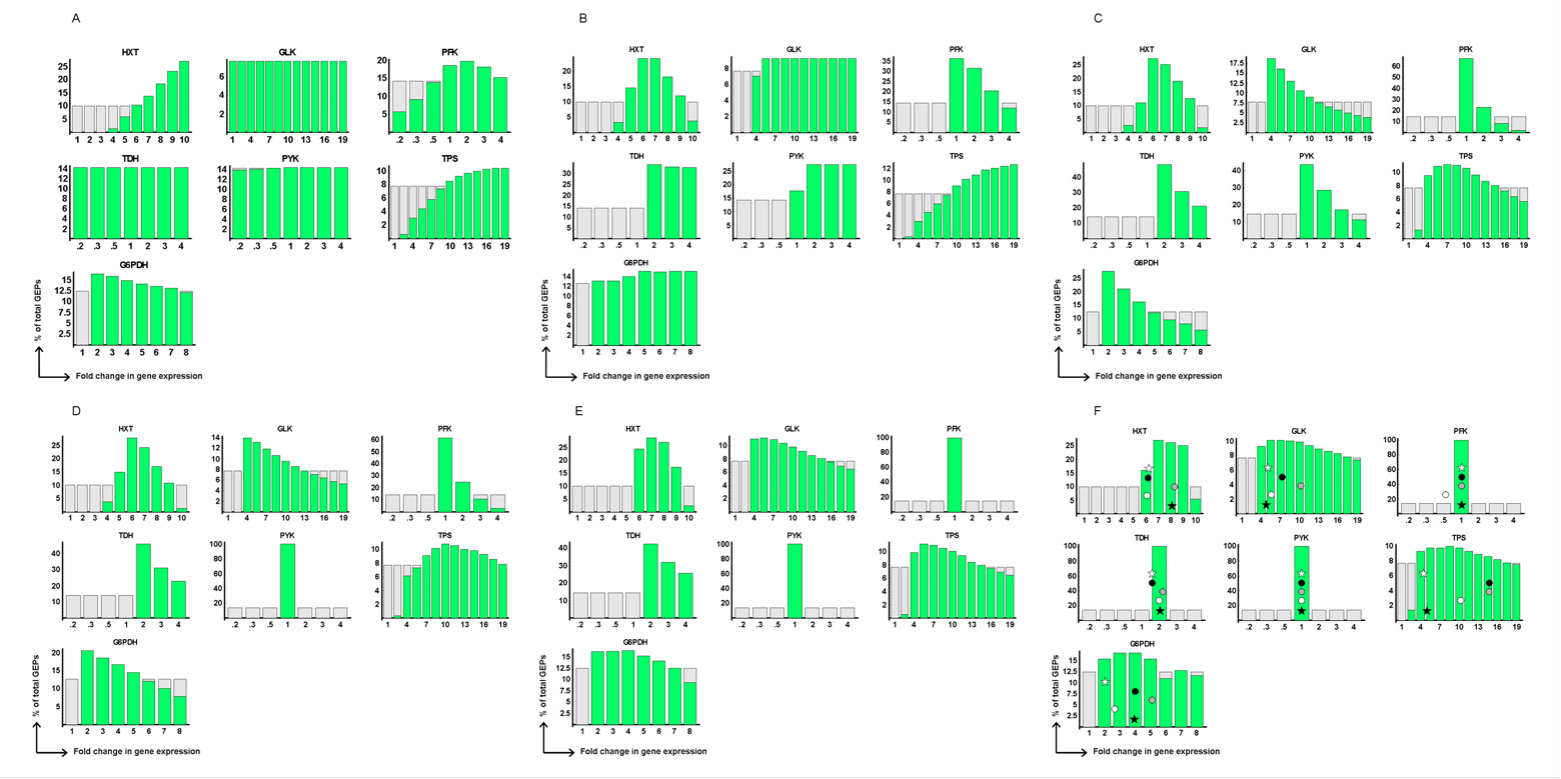
**Cumulative selection of GEPs by sequential application of the functional criteria**. Constraints to gene expression resulting from the application of the different criteria. The x-axis represents the fold-change in gene expression with respect to the basal levels for the different genes. The y-axis represents the percentage of GEPs that have a given fold-change in gene expression. Light grey bars represent the distribution of gene expression in GEPs prior to any selection. This distribution is uniform. Green bars represent the distribution of fold change in gene expression in the set of GEPs that obey a given combination of criteria. This distribution is superimposed to the prior distribution, showing how the criteria constrain the expression of different genes. Panels show results of applying several criteria in combination: **A **- C1-C3,**B **- C1-C4, **C **- C1-C5, **D **- C1-C6, **E **-C1-C7, and **F **- C1-C8. Changes in the levels of expression for each gene from the different experimental datasets are represented as discrete points in panel F. Gene expression changes for different genes from the same dataset are shown at the same height in each row of individual plots. Black stars – data from Eisen's experiments at 10 min (BD1 10'); White stars – data from Causton's experiments at 15' (BD2 15'); White circles – data from Gasch's experiments at 10' (DB3 10'); Grey circles – data from Gasch's experiments at 15' (DB3 15'); Black circles – data from Gasch's experiments at 20' (DB3 20').

In the past, constraints to metabolic fluxes have been used as the main physiological criteria for deciding about the appropriateness of gene expression changes in response to different types of stress. Different mathematical formalisms, such as Flux Balance Analysis (see e.g. [99] for a review), linear formalisms [48] and Michaelis-Menten like formalisms [26] are the basis of the mathematical models that are used to evaluate those constraints. However, there is an accumulating body of work showing that aspects other than flux, such as concentrations and tolerances, play an important role in explaining the system's adaptation to environmental changes (e.g. [29,100]). Our analysis also shows that flux based functionality criteria can not fully explain the pattern of changes in gene expression that are observed after heat shock. The flux criteria only constrain the expression of genes that directly increase the flux of ATP synthesis (HXT), trehalose synthesis (TPS) and NADPH (G6PDH) synthesis (Fig. 2A). Thus, specific changes in the expression of other genes must be explained using additional physiological criteria that are not flux based.

### Additional criteria for functional effectiveness in response to heat shock

The first three criteria discussed above place metabolic flux-based constraints on the changes in gene expression that yeast uses to adapt to heat shock. However, although these constraints are clearly important and necessary, they are by no means sufficient to account for all the GEPs changes observed in response to heat shock. Other physiological constraints must be considered in order to understand the design principles of the GEP response to heat shock. Because these additional constraints are not explicitly flux dependent, they can not be analyzed using tools such as flux balance analysis in its present forms. Our approach, based on a GMA mathematical model provides a practical tool for evaluating such complementary criteria. Based on preliminary work about the identification of design principles in metabolic systems (see e.g. [101] and references therein), we have considered three additional criteria that regard metabolite accumulation of metabolites and economy of gene expression and protein synthesis:

**C4. Changes in gene expression should allow cells to avoid needless increases in the concentration of intermediates upon heat shock. **Buffering the concentration of different intermediates is needed to limit cross-reactivity, avoid cell solubility problems, and prevent metabolic waste (see e.g [102,105]). Although a minimal increase in the concentration of intermediates is an appropriate criterion, some key metabolites are not just intermediates; they have a function. The concentration of these "functional" metabolites must change to accommodate the changes in metabolic functions. The cut-off values for the different metabolites considered by our model are set according to the following criteria:

a. ATP concentration should increase to meet energy demands. Although we found no accurate measurements for ATP concentrations after heat shock, indirect observations can be used to define a reasonable cut-off value. Heat shock chaperone proteins are needed to fight protein denaturation and aggregation after heat shock (see [106,107] for reviews). Heat shock specific chaperones are active at high ATP concentrations [108]. *In vitro *studies show that chaperones are active at more than 90% of their maximal specific activity under ATP concentrations of 5 mM or more [108,109], which are at least 5 times higher than concentrations at the basal steady state. Allowing for an additional one fold increases in ATP concentration due to other processes such as an increase in energy demand, we estimate that a six fold increase in ATP concentration as sufficient for appropriate heat shock response. Consequently, GEPs that have an increase in the concentration of ATP higher than six fold are eliminated.

b. After heat shock, Winkler [93] found increases of about an order of magnitude in the concentrations of both G6P and UDP-glucose. These metabolites are needed for energy production. Because, in our model the variable G6P lumps G6P with UDP-glucose, a cut-off of 20 over the basal level for this lumped variable seems appropriate to capture the physiological response. Thus, profiles in which G6P concentrations increase by more than 20 times are eliminated.

c. The concentration of fructose-1,6-biphosphate (F16P) must increase because TPS is less sensitive to activation by this metabolite under heat stress [110-112]. Given that TPS is fully active *in vitro *at F16P concentrations higher than 10 mM [111] and the basal concentration of F16P in the model is about 9 mM, the concentration of F16P is allowed to increase at most 2.5-fold in order to allow for this activation. Additionally F16P activates several other fluxes whose up-regulation is critical for an adequate heat shock adaptation (see also criteria C6 and C8).

d. We have found no estimates for the heat shock concentrations of the remaining metabolites. As they have no known specific role in heat shock response, we choose a maximum increase of a 20% over the basal concentration of each remaining metabolite as the cut-off value for the criteria. This threshold value was found to be small enough to accommodate unavoidable increases in concentration due to increases in flux. Thus any profile in which the concentration of each of the remaining metabolites increases by more than 1.2 times is eliminated.

**C5. Adaptation should be economic. **GEPs that allow adaptation with minimal changes in gene expression should be favoured [57], among other things because they minimize protein burden to the cell [113,114]. There is little quantitative information about metabolic cost of protein synthesis. Therefore, we choose to use the simplest measure that captures the overall cost of the process. We use the sum of the logarithmic change of expression for all enzymes gives a measure of the cost of changing gene expression [27]. We have also performed some preliminary analysis using different cost functions that, among other aspects, weight the cost of protein synthesis with protein length and amino acid metabolic cost. The preliminary results show that no major differences are observed in the final results. This will be discussed in detail in a paper in preparation (unpublished results). We found no available experimental information to estimate this criterion's cut off value. As a first approximation for the cut-off value we used an internal standard to the set of GEPs. We choose the cut-off value to be the integer value closest to the fiftieth percentile of the set of profiles, resulting in a cut-off value of 12.06.

**Figure 3:**
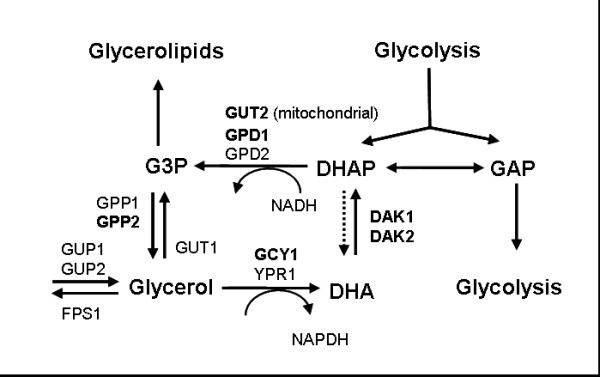
**Pathway and genes that are involved in the metabolism of glycerol**. Bold genes are considered to be over-expressed. Abbreviations: GAP, glyceraldehyde 3-phosphate; G3P, glycerol 3-phosphate; DHAP, dihydroxyacetone phosphate; DHA, dihydroxyacetone; GUT2, mitochondrial glycerol-3-phosphate dehydrogenase 2; GPD1, glycerol-3-phosphate dehydrogenase 1; GPD2, glycerol 3-phosphate dehydrogenase 2; DAK1, dihydroxyacetone kinase 1; DAK2, dihydroxyacetone kinase 2; GPP1, DL-glycerol-3-phosphatase 1; GPP2, DL-glycerol-3-phosphatase 2; GUP1, glycerol uptake protein 1; GUP2, glycerol uptake protein 2; GUT1, glycerol kinase 1; FPS1, plasma membrane glycerol channel; GCY1, putative NADP^(+) ^coupled glycerol dehydrogenase; YPR1, GCY1 homolog.

**C6. GEPs with the highest glycerol accumulation after heat shock are favoured. **Glycerol is used to maintain the integrity of cellular components [115] and the metabolic cycle responsible for the biosynthesis of glycerol (Fig. 3) converts NADH into NADPH [116,117]. Most of the genes coding for enzymes of this cycle are over-expressed under heat shock conditions (Table 3). Part of the accumulation effect in the glycerol pool during stress conditions is due to down-regulation of glycerol extra cellular export [118]. Due to lack of kinetic data our model can not account for the change in export rates. However, we can use the predicted rate of glycerol synthesis through the cycle in our model as a proxy for the increase in glycerol concentration. To define a cut-off value for the proxy we need to use an internal standard of the GEP population. We set that value at 0.39 mM min^-1^, which is the value at which 50% of GEPS are selected. Any GEP that has a glycerol production flux that is lower that this value is eliminated.

Applying criterion C4 on the GEPs resulting from the first three criteria reduces by a factor of ten the number of GEPs that lead to an appropriate cellular response (111391 selected cases, 2.40%, Fig. 3B). According to this criterion, GEPs where the gene expression of PFK and PYK is repressed upon heat shock do not lead to appropriate cellular adaptation. Repression of PFK mainly cause accumulation of glucose-6-phosphate or insufficient flux of ATP. Respression of PYK mainly causes accumulation of phosphoenolpyruvate. This criterion excludes GEPs that have an increase in the activity of GLK of less than four times that of the basal state. Under this threshold, glucose accumulates and no compensation can be achieved by changing other enzymes. An increase in the gene expression of TDH that is smaller than two times that of the basal state is also excluded by this criterion because fructose-1,6-biphosphate concentrations rise over the cut-off. Finally, this criterion significantly decreases the maximum allowable change in activity for HXT. For example, less than 5% of GEPs that fulfil this criterion have an increase in HXT activity higher than ten times. This is the most stringent criteria of all.

Application of criteria C5 on the results of C1-C4 reduces the number of GEPs that fulfil these physiological criteria (27227 selected cases, 0.50%). GEPs selected under this criterion (Fig. 3C) have an average decrease in the over-expression of all enzymes in comparison to the profiles selected by the first four criteria.

**Figure 4:**
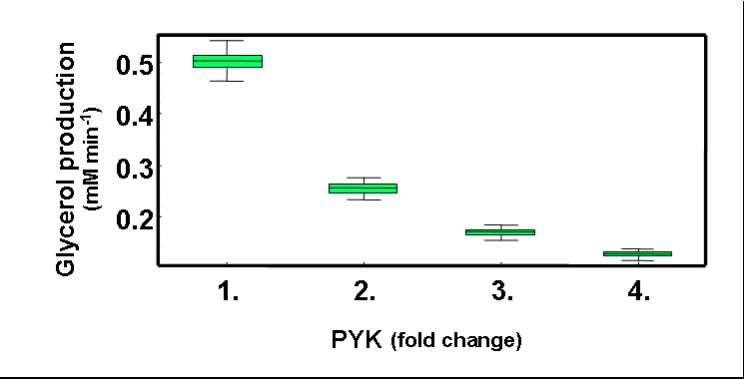
**PYK over-expression and glycerol flux**. Box and whisker plots show correlation between trehalose production and changes in the gene expression of PFK and TPS. Each entry in the box-and-whisker plot is represented by a box that spans the distance between two quantiles (0.25 and 0.75) surrounding the median. The median is shown by a horizontal line crossing the green box. "Whiskers" lines extend to span the full range of values for that entry.

Criteria C6 applied to the results of C1-C5 further reduce the number of GEPs by a factor of two (0.25% of all GEPs are selected, Fig. 2D). Additional selection by this criteria imposes an upper limit to the gene expression of PYK that is lower than that observed in GEPs selected based on the five previous criteria. This occurs because over-expression of PYK is inversely correlated to the rate of glycerol synthesis (Fig. 4). Thus, GEPs with a large increment in the gene expression of PYK prevent adequate adaptation to heat shock because these GEPs prevent the cells from producing enough glycerol.

**Figure 5:**
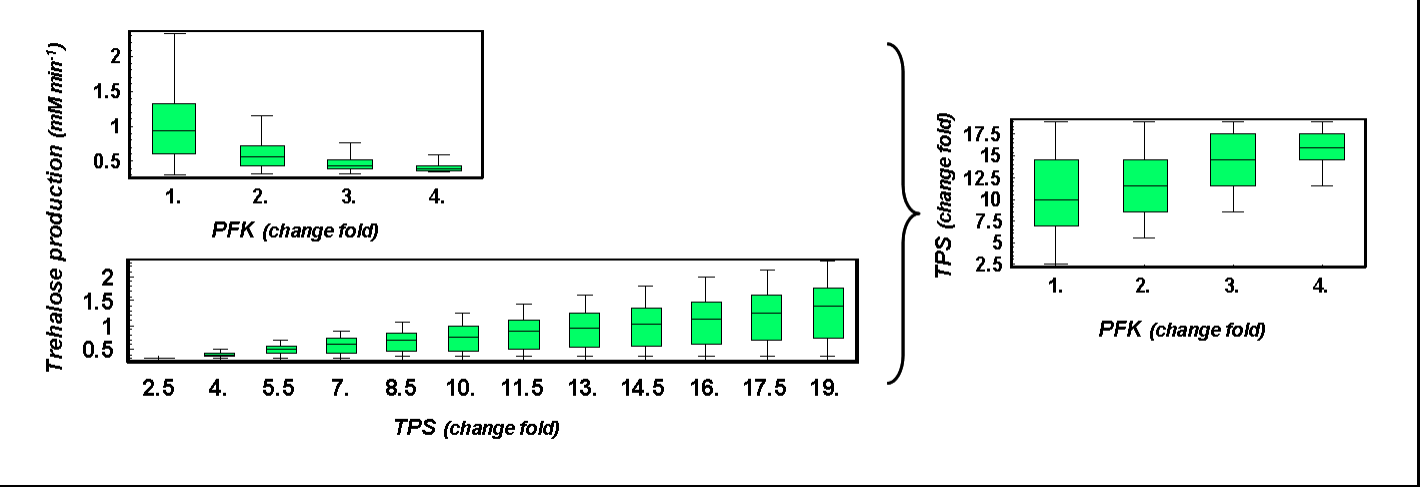
**PFK and TPS over-expression**. The production of glycerol and the activity of pyruvate kinase are strongly coupled in GEPs that are well adapted for heat shock response. The x-axis represents the fold-change in PYK gene expression. The y-axis presents the production of glycerol. High expressions of PYK lead to lower production rates of glycerol. This constrains the fold-change in PYK gene expression, because a minimum rate of glycerol production is necessary for adaptation to heat shock.

Analysis of the GEPs resulting from seleccion by C1-C6 suggests two new functional constraints that refine the previous set of criteria:

**C7. Changes in the activity of the enzymes TPS and PFK should be co-ordinately balanced after heat shock. **Trehalose levels are determined mostly by the action of these two enzymes. TPS catalyzes the reaction that produces trehalose, and any increase in the activity of this enzyme is directly correlated with the level of that metabolite. On the other hand, PFK drives flux away from trehalose production and into the production of ATP, although over-expression of this enzyme does not directly correlate with high concentrations of ATP (data not shown). Thus, an increase in the expression of PFK requires a compensatory increase in the expression of TPS in order to maintain the flux of trehalose synthesis (see Fig. 5). Due to this coordinated requirement one expects that an adequate GEP should have a parsimonious change in the gene expression for both enzymes coordinated with an increase in the productive flux of trehalose. We use the index Ψ = (Δ*TPS *× Δ*PFK*)/*ν*_*trehalose *_as a measure for this criterion. In this index Δ*TPS *is the change in TPS gene expression, Δ*PFK *is the change in PFK gene expression, and *ν*_*trehalose *_is the flux of trehalose production. All changes are measured with respect to the basal steady state. A small value for this index ensures that the changes in TPS and PFK are parsimonious and that they provide an appropriate trehalose production. Thus, only GEPs in which the value for this index is low are selected as being able to lead to proper adaptation to heat shock. To be consistent in selecting a numerical value for this criterion, we used the same approach described for C6. From the set of GEPs that numerically fulfil criteria C1-C3, we selected the 50% of cases with the lowest value for Ψ. The highest value for Ψ in that set was then used as a cut-off value for C7 throughout our study. This new criterion may be seen as a refinement complementary to C5.

**C8. Changes in gene expression should allow the cells to avoid depletion of F16P after heat shock. **F16P is needed for the production of glycerol and has a feed-forward activation effect on the enzymes of the lower part of glycolysis. *In vitro*, the rate of the reaction catalyzed by PYK is up to twenty times higher in the presence of F16P and hexose phosphates at their physiological concentration ranges than in the absence of these metabolites [119]. This modulation of flux facilitates the flow of material and avoids accumulation of intermediates. Thus, lack of F16P may hinder cellular adaptation to heat shock. This suggest discarding any GEP in which the concentration of F16P was less than 95% that of the basal state. This criterion is complementary to C4. While C4 is used to prevent needless accumulation of intermediates, C8 ensures that a needed intermediate (F16P) is not excessively depleted thus preventing adaptation to stress.

Application of criteria C7 on the results from criteria C1-C6 reduces the number of GEPs only slightly (7295 cases 0.16%, Fig. 2E). Profiles selected using criterion C7 have changes in the activity of HXT that are larger than or equal to 6 times the basal activity of this enzyme, thus increasing on average by almost 50% the minimum activity allowed by criteria C1-C3. Additionally, the activity of PFK should not change.

Application of criteria C8 on the GEPs selected by C1-C7 reduces the number of acceptable GEPs by half (2935, 0.06% ). Criterion C8 sharply constrains the allowable changes in the expression of TDH (Fig. 2F). Only GEPs where the gene expression of TDH is 2-fold that of the basal state can adequately adapt to heat shock.

### Model predictions and observed gene expression profiles after heat shock

Our results can be compared with actual microarray data in the following way. For each available dataset, we took the fold change in the expression of the genes present in our model at the relevant time points and using those values we calculated the corresponding changes in enzyme activity (Fig. 2F and Table 1). This was done as described for the *in silico *profiles. We then verify if the experimentally derived GEPs are among the ones that passed all our criteria for an effective cellular adaptation to heat shock (Table 4). Overall, the gene expression changes observed in each of the experimental datasets match the changes predicted by our *in silico *analysis as providing an appropriate physiological adaptation to heat shock.

Only the predicted changes to the gene expression of TDH and PFK did not match the experimental observations. These discrepancies may have two origins. On one hand, our model may not be sufficiently detailed. On the other, the noise in the datasets may be large enough to account for the discrepancies. Our results predicted that a 2-fold increase in the gene expression of TDH was needed in response to heat shock in order to allow for an adequate cellular adaptation. The *in silico *scanning of the changes in gene expression only performed a discrete sampling of values for the activity of TDH. Thus, we did not test increases in the gene expression of this enzyme that were between one and 2-fold that of the basal gene expression. The experimental results showed increases of gene expression of 1.3 at BD2 15' and of 1.4 at BD3 20'. To better characterize this problem, we refined our search and tested *in silico *GEPs that considered changes in the expression of TDH that were between no change and twice the basal level of expression. We found that, according to our criteria, these profiles also allowed the cells to adequately adapt to heat shock. The TDH changes that were observed in BD2 15' and BD2 20' were contained in the new *in silico *profiles that were selected as allowing appropriate cellular adaptation to heat shock. Thus, the discrepancy between experimental results and theoretical analysis was removed by a more detailed *in silico *analysis.

Our *in silico *results also predict that the gene expression of PFK must remain almost unchanged for an adequate cellular adaptation to heat shock. This is observed in four out the five experimental datasets. However, the experimental results from BD3 10' showed a 2-fold repression in the gene expression of PFK after heat shock. *In silico *profiles with this change were excluded by our functional criteria as not allowing cells to adapt adequately to heat shock. To explore if a 2-fold decrease in gene expression could be a result of experimental noise, we complement our analysis in the following way. For each experiment we measured the quantiles for the change in gene expression for all the genes that were present in the microarrays. Table 5 shows the change-fold obtained by comparing pre-stressed cells with themselves. In this situation change-fold values should be 1, because no difference is expected between different samples of the same population. Observed values that are different from 1 are due to experimental noise. The upper bound for this noise is around twice the basal level of gene expression (0.95 quantile) and the lower bound is around half the basal value of gene expression (0.05 quantile). Thus a 2-fold decrease in gene expression can be due to experimental noise. These results suggest that the observed repression of PFK in BD3 10' does not contradict our prediction within the precision of the available experiments.

**Figure 6:**
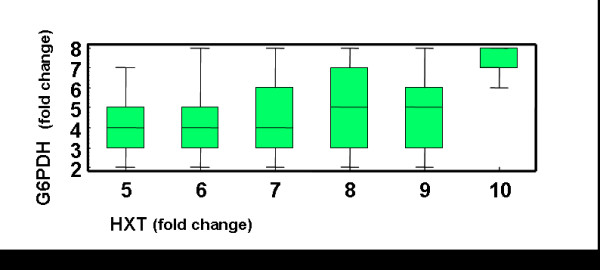
**G6PDH and HXT over-expression**. Box and whisker plots (see Fig.5 legend for further details) showing the joint distribution of the changes in the expression of G6PDH and HXT in the GEPs that fulfil all the performance criteria.

Fig 2F shows that activities of four of the enzymes have a narrow change and that the activities of the remaining enzymes have to increase. For some of the genes that are over-expressed, our criteria allow for a large range of over-expression, with the microarray derived sets being in or next to the modal groups. One interpretation for this data is that our criteria are sufficient to justify most of the changes in gene expression observed on the microarray data, at least semi-quantitatively. For some of the enzymes the spread seen on the panels of Figure 2F is larger than that observed in microarray data. A particular example of this situation is the combination of high expression values of HXT and G6PDH (Figure 6). According to our analysis, it is theoretically possible to have over-expression of HXT over 10-fold only if G6PDH is over-expressed at least 6-fold. Otherwise, the cells would accumulate G6P and PEP at concentrations that are higher than the cut-off values allowed by criterion C4. However, there are no experimental cases with change-fold values that are as high as these. The larger spread of the allowed ranges for GLK, TPS and G6PDH in the selected GEPs can reflect a combination of the following three situations. i) Cells can over express these genes over a range and still properly adapt to heat shock. This could reflect a lower evolutionary pressure that leads to a larger variability of the changes in the expression of these genes. ii) The cut off values for the criteria need to be more stringent. For instance, an increase in the stringency of criterion C4 decreases the spread of the allowed ranges for GLK, TPS and G6PDH in the selected profiles (data not shown). iii) Additional criteria should be considered to understand why the predicted spread of some genes is larger than the observed in the microarray data. However, our analysis gives no indication that this may be the case.

Development of measurement techniques that reduce noise in the data [120-122] and methods for microarray data normalization that improve the sensitivity to the signal [123] will improve the quality of the available experimental data. However, the qualitative aspects of our predictions are robust to small fluctuations of the exact experimental values observed for the transcriptional changes. Having very precise experimental measurements would help in refining the cut-off values of the considered constraints, which could more accurately elucidate the quantitative design principles of gene expression in the adaptive response. Furthermore, accurate measurements for the early dynamics of gene expression after heat shock at many different time points would help in determining functional dynamic constraints that would expand the set of steady state constraints used in this work. Work in progress in our lab is addressing both these issues.

### Final remarks

To summarize the results, only a small fraction (0.06%) of all tested GEPs leads to a proper response to heat shock, as defined by all eight criteria for functional effectiveness. Analysis of the selected GEPs reveals a profile that has well-defined characteristics. HXT should be over-expressed at least by a factor of 6, and values higher than 9 are not favoured. GLK should also be over-expressed, at least by a factor of 4, and no functional reasons are found to avoid significantly large increases. PFK gene expression should not change. The performance criteria require a 2-fold increase for TDH, and no change in the expression levels of PYK. Finally at least an over-expression of 2.5-fold for TPS and a 2-fold for G6PDH are also required. Overall, even after allowing for gene expression changes that have several fold difference from the experimental GEPs, after applying the functionality criteria, only GEPs that are very similar to the experimentally observed ones provide an appropriate response to heat shock.

A more detailed analysis of the functional effectiveness criteria (paper in preparation) shows that the set of criteria defined here is very specific for heat shock response. We implemented into the model the transcriptional changes from experimental data caused by all the environmental changes reported in the three experiments. Finally 297 different GEPs from databases were implemented in the mathematical model. This number includes some repeated conditions, oxidative, osmotic, acid and basic stress, etc. Our set of criteria only selected the heat shock conditions, the previously used to explore the adaptive response, and a new GEP that comes from DB3, transcriptional changes at 20 minutes after a temperature shift from 29 to 37 °C. These encouraging results strongly suggest that the set of criteria constitute an adequate and specific definition of performance of cells to heat shock.

A final word must be said about the dynamics of the response to heat shock. As some studies shown, consideration of the dynamic response may help in devising some important features of the design principles underlying the systemic response to environmental changes [25]. Although this analysis is possible and desirable, not enough data is available about the temporal changes of the gene expression in response to heat shock. This is so because the response to heat shock in yeast induces quick changes in expression, that occurs around 10 minutes after the shock and are mostly completed 20 minutes after heat shock. For all published microarray experiments, there are few data points in this critical time interval. We analyzed the temporal response of some of the selected profiles and found a large variability of the time response. This further supports the notion that more data is available before time dependent criteria are considered.

## Conclusion

This paper presents a case study in which heat shock response in yeast is analyzed to determine quantitative design principles that constrain changes in gene expression. This work relies on combining theoretical and mathematical methods to analyze the physiological effects of hypothetical adaptive responses. This provides a framework to test evolutive implications of gene expression changes, based on evaluating the performance of the different GEPs. By combining theoretical methods and mathematical models we present an integrated approach that can help in understanding the observed adaptive response in terms of precise quantitative design principles.

We were able to identify eight criteria for physiological effectiveness that quantitatively constrain the heat shock response at the genetic level. The criteria that account for increased production of ATP, trehalose and NADPH are the ones that have more extensive experimental support. However, our work shows that these criteria are not the only determinants that constrain gene expression changes in response to heat shock. Five additional criteria for functional effectiveness that constrain gene expression have been identified. These additional criteria include constraints to other productive fluxes of protective molecules, to the physiological concentration of the different metabolites, and considerations about the metabolic economy of the response. Only 0.06% of all tested GEPs lead to proper heat shock adaptation, according to our model and criteria.

When GEPs from microarray experiments are analyzed using the model, we find that they correspond largely to *in silico *profiles that allow cells to adapt to heat shock according to all eight criteria. Exceptions are the levels of change in the expression of the genes coding for PFK. When we refine our theoretical analysis and account for noise in the data, all experimental profiles fit our *in silico *predictions. Thus, the theoretical analysis is validated by comparison to data from microarray experiments. Our results indicate that yeast has evolved a very precise adaptive response to heat shock and they suggest that this response is largely constrained by physiological requirements. Our predictions extend previous results on this problem [27] through extensive computation and evaluation of alternative GEPs.

The theoretical approach suggested in this work has also some potential limitations. First, as models are simplifications of the biological situation, one should not expect that they account for all details of the system being modelled. Despite this, our model captures many of the metabolic features that are known to be characteristic of the yeast adaptation to heat shock. Our results show that the level of detail used in the current model is enough to match the experimental observations and to identify quantitative design principles explaining the experimental outcome. Second, the precise change-fold values vary from one database to another due to intrinsic variability of the experimental methods and conditions. However, when the observed GEPs are introduced in the model the corresponding changes in enzyme levels produce similar qualitative physiological responses. Accordingly, these can be used as guidelines for the proposed analysis despite the experimental uncertainty.

More precise experimental determination of actual GEP in response to stress conditions would lead, at the best, to a better descriptive picture of yeast adaptation. But even a precise determination of this profile would not explain why it was selected among other possibilities. Our work provides clues as to which criteria for functional effectiveness are more important in determining the changes in the individual genes during heat shock response. It also provides a global, semi-quantitative, picture of the pattern of gene expression changes that one should expect as a response to heat shock stress. The methods and biological results presented in this paper provide clues that help in understanding how physiological constraints corset changes in gene expression in response to environmental cues. An application of the methods to analyze other types of stress is bound to reveal the commonalities and specificities of different types of stress response at the level of the changes in gene expression. This analysis may be less straightforward in other instances because the coupling between changes in mRNA levels and changes in enzyme activity may be more complex. Nevertheless, applying our approach to analyze such cases could be of great interest in predicting potential targets for post-transcriptional regulation of activity. This will greatly contribute to the understanding of adaptive responses at the cellular level.

## Methods

### Mathematical model of the pathways involved in heat shock response of yeast

To build our model, we use the Generalized Mass Action (GMA) [27,124-126] canonical within the power-law formalism. In this form, the differential equations for each of the *n *dependent variables are represented in the form:

dXi/dt=∑j=1r(μi,jαj∏k=1n+mXkfj,k)i=1,…,n,
 MathType@MTEF@5@5@+=feaafiart1ev1aaatCvAUfKttLearuWrP9MDH5MBPbIqV92AaeXatLxBI9gBaebbnrfifHhDYfgasaacH8akY=wiFfYdH8Gipec8Eeeu0xXdbba9frFj0=OqFfea0dXdd9vqai=hGuQ8kuc9pgc9s8qqaq=dirpe0xb9q8qiLsFr0=vr0=vr0dc8meaabaqaciaacaGaaeqabaqabeGadaaakeaafaqabeqacaaabaGaemizaqMaemiwaG1aaSbaaSqaaiabdMgaPbqabaGccqGGVaWlcqWGKbazcqWG0baDcqGH9aqpdaaeWbqaamaabmaabaacciGae8hVd02aaSbaaSqaaiabdMgaPjabcYcaSiabdQgaQbqabaGccqWFXoqydaWgaaWcbaGaemOAaOgabeaakmaarahabaGaemiwaG1aa0baaSqaaiabdUgaRbqaaiabdAgaMnaaBaaameaacqWGQbGAcqGGSaalcqWGRbWAaeqaaaaaaSqaaiabdUgaRjabg2da9iabigdaXaqaaiabd6gaUjabgUcaRiabd2gaTbqdcqGHpis1aaGccaGLOaGaayzkaaaaleaacqWGQbGAcqGH9aqpcqaIXaqmaeaacqWGYbGCa0GaeyyeIuoaaOqaaiabdMgaPjabg2da9iabigdaXiabcYcaSiablAciljabcYcaSiabd6gaUjabcYcaSaaaaaa@6064@

where *X*_*i *_represents the concentration of metabolite *i *in the model. The model has *m *independent variables that will be considered as parameters, and *r *fluxes that contribute to the change in the concentration of the pool of *X*_*i*_. *μ*_*i*, *j *_is the stoichiometric coefficient; it is positive if the flux *j *produces *X*_*i *_and negative if the flux *j *depletes the pool of *X*_*i*_. *α*_*j *_is an apparent rate constant for flux *j*. *f*_*j*, *k *_is the kinetic order of variable *X*_*k *_in reaction *j*. Each kinetic order quantifies the effect of the metabolite *X*_*k *_on flux *j *and corresponds to the local sensitivity of the rate *ν*_*j *_to *X*_*k *_evaluated at the operating point indicated by the subscript 0:

fj,k=(∂νj∂XkXkνj)0
 MathType@MTEF@5@5@+=feaafiart1ev1aaatCvAUfKttLearuWrP9MDH5MBPbIqV92AaeXatLxBI9gBaebbnrfifHhDYfgasaacH8akY=wiFfYdH8Gipec8Eeeu0xXdbba9frFj0=OqFfea0dXdd9vqai=hGuQ8kuc9pgc9s8qqaq=dirpe0xb9q8qiLsFr0=vr0=vr0dc8meaabaqaciaacaGaaeqabaqabeGadaaakeaacqWGMbGzdaWgaaWcbaGaemOAaOMaeiilaWIaem4AaSgabeaakiabg2da9maabmaabaWaaSaaaeaacqGHciITiiGacqWF9oGBdaWgaaWcbaGaemOAaOgabeaaaOqaaiabgkGi2kabdIfaynaaBaaaleaacqWGRbWAaeqaaaaakmaalaaabaGaemiwaG1aaSbaaSqaaiabdUgaRbqabaaakeaacqWF9oGBdaWgaaWcbaGaemOAaOgabeaaaaaakiaawIcacaGLPaaadaWgaaWcbaGaeGimaadabeaaaaa@449C@

If *X*_*k *_has no direct influence on the rate of reaction *j*, the kinetic order is zero. If *X*_*k *_directly activates the flux of reaction *j*, the kinetic order is positive. If *X*_*k *_directly inhibits the flux of reaction *j*, the kinetic order is negative.

The model we study is a version of the biosynthesis of glycerol, trehalose, and glycogen and the first step of the pentose phosphate pathway (Fig. 1). It was originally published and analyzed by Curto and colleagues [37-39]. In that publication parameter estimates are justified, sensitivity analysis of the model is also performed and the dynamic behaviour of the model is studied. Voit and Radivoyevitch [27] extended this model by adding two branching pathways that are mathematically represented in S-system form: i) the first reaction of the pentose phosphate pathway and ii) the first reaction of the biosynthesis of trehalose. Again, the parameter estimations for the added reactions are fully explained in Voit and Radivoyevitch's publication [27]. We use this model with the same parameter values but translating the original S-system model into a GMA form. The S-system representation aggregates fluxes that contribute to the increase in the concentration of a given metabolic pool into one single black box process and the fluxes that contribute to the decrease in the concentration of a given metabolic pool into another single black box process [see [70] and references therein]. As per our functionality criteria, we needed to consider the fluxes of the individual processes. Converting from the S-system into the GMA representation allows the analysis of the individual fluxes. The precise relationship between GMA parameters and S-systems parameters has been discussed elsewhere [see for instance [38,70]]. Mathematical details about the model implementation can be found in the referred publications or by reading the SBML description that can be downloaded from the web [127]. All computations were done using Mathematica™ [128] and the open-source package MathSBML [129].

### Generation of hypothetical adaptive scenarios

To study the effect of gene expression changes on yeast adaptation to heat shock we proceeded in the following way. First, we analyzed the mathematical model using parameter values and enzyme activities that correspond to the basal, pre-stress situation (25°C). Second, we implemented stress related changes by considering that the gene expression changes caused by heat shock correspond to changes in the enzyme activities of the model. Third, the physiological effect of each expression profile was calculated by changing the basal enzyme activity level in accordance with the fold-change in gene expression. We only found evidence in the literature for post-transcriptional activation of TDH and PYK [27,85,86]. Thus these were the only enzymes in our model for which we considered post-transcriptional modulatory effects. Finally, we characterized the new steady state resulting from each GEP. Each new steady state is compared to the basal steady state. The differences were analyzed in the context of the known physiological response of the cells to stress. If the new steady state did not pass criteria for adequate functionality (see Results for a description of the criteria) then the GEP that generated this steady state was not considered to generate a viable response to heat shock.

The scanning of physically plausible values for *in silico *gene expression changes was done as follows. We assumed that expression of each gene could change within the ranges described in the caption for Fig. 1. These ranges where selected after analyzing the fold changes for the considered genes reported in the available microarray data. By default, for each gene, a range of several fold-changes above and below its pre-stress basal state was scanned. This range always includes and surpass by at least two-fold units the largest change observed in microarray data. In some cases (see Fig. 1) this scanning was truncated because either over-expression or under-expression of the relevant gene is excluded by known physiological information. For such cases sampling was extended by a few folds in the direction that was not truncated. Within the corresponding ranges for each gene, we scanned the expression for the genes corresponding to the enzymes in the model. Each combination produces a particular GEP. We combined all allowable values for the expression of each gene, leading to the initial analysis of 4,637,360 *in silico *GEPs.

### Available microarray data characterizing heat shock response in yeast

The GEPs corresponding to the response of yeast to heat shock have been investigated by several groups [3-5]. The experimental GEPs available present data from three independent experiments in which yeast cells were subjected to heat shock. In each independent experiment cells were collected at different times after the temperature jump. The three experiments reveal a similar dynamic of gene expression, where maximal changes in gene expression with respect to pre-stress levels are observed between 10 and 20 minutes after heat shock. Afterwards, expression of most genes returned to values that are comparable to pre-stress levels. If we focus on the peaks of gene expression changes, this corresponds to 10 minutes after heat shock (DB1 10') in Eisen's experiments [3], 15 minutes after heat shock (DB2 15') in Causton's experiments [5], and 10 (DB3 10'), 15 (DB3 15') and 20 (DB3 20') minutes after heat shock in the data obtained in Gasch's experiments [4].

## Abbreviations 

BST, Biochemical Systems Theory; SBML, Systems Biology Markup Language; GMA, Generalized Mass Action model; TDH, glyceraldehyde-3-phosphate dehydrogenase; PYK, pyruvate kinase; GEP, Gene Expression Profile; BD1, data from Eisen's experiments [3]; BD2, data from Causton's experiments[5]; DB3, data from Gasch's experiments [4]; G6P, glucose-6-phosphate; F16P, fructose-1,6-biphosphate;TPS, trehalose 6-phosphate syntase complex; PFK, phosphofructokinase; SGD, Saccharomyces Genome Database; HXT, hexose transporters; G6PDH, glucose 6-phosphate dehydrogenase; GLK, glucokinase/hexokinase; PEP, phosphoenolpyruvate.

## Authors' contributions

EV participated in the conception of the study, carried out the programming and in the drafting of the manuscript. RA participated in the design of the study and in the drafting of the manuscript AS initially conceived the study, participated in its design and computer programming, its coordination, and in the drafting of the manuscript. All authors read and approved the final manuscript.

## Figures and Tables

**Table 1:** Values of criteria for functional effectiveness calculated using the GEPs derived from the published experimental datasets.

Experimental Dataset							
		Basal	DB1 10' ^a^	DB2 15' ^b^	DB3 10' ^c^	DB3 15' ^c^	DB3 20' ^c^

Gene expression fold-change with respect to pre-stress situation	HXT	1	8	6	5.6	8.6	5.8
	GLK	1	4.8	5.2	5.7	10.5	7.2
	PFK	1	1	0.9	0.5	0.8	0.9
	TDH	1	1.8	1.3	1.7	2.2	1.4
	PYK	1	1.1	1.2	1	1	1
	TPS	1	5	4	9.9	15.6	15.7
	G6PDH	1	3.8	1.8	2.4	5	3.9

Fluxes^d^	V_ATP_	60.2	263.8	222.4	175.0	262.6	199.4
	V_TRE_	0.0012	0.0535	0.0343	0.1202	0.1830	0.1213
	V_NADPH_	1.8	7.7	3.7	4.9	10.3	7.7

a: BD1 10': data from Eisen's experiments at 10 min. b: BD2 15': data from Causton's experiments at 15'. c: DB3 10', DB3 15' and DB3 20': data from Gasch's experiments at 10', 15' and 20', respectively. d: Rate of synthesis (mMmin^-1^).

**Table 2:** Selection of GEPs using the criteria for functional effectiveness.

	Values for Criteria		Percentage of GEPs selected using each criteria	
	Absolute values	Ratio to basal values	Individual	Accumulated

**C1**	V_ATP_^a ^> 180.6	3	45.13^e^	
**C2**	V_TRE_^a ^> 0.03	25	60.95^e^	
**C3**	V_NADPH_^a ^> 3.54	2	85.86^e^	27.83
**C4**	GLC^b ^< 0.04	1.2	86.40^f^	
	G6P^b ^< 20.22	20	76.04^f^	
	F16P^b ^< 22.86	2.5	51.91^f^	
	PEP^b ^< 0.01	1.2	65.44^f^	
	ATP^b ^< 6.77	6	89.32^f^	2.40
**C5**	Cost^c ^< 12.06	12.06	50	0.59
**C6**	V_Glycerol_^a ^> 0.39	0.22	50	0.25
**C7**	Ψ^d ^< 28.10	0.391	50	0.16
**C8**	F16P^b ^> 8.64	0.95	61.93	0.06

(a) Rate of synthesis (mMmin^-1^). (b) Concentration (mM). (c) Adimensional. (d) mM^-1^min. (e) Application of C1-C3 selects 27.83% of all GEPs. (f) Application of constraints on all concentrations simultaneously selects 24.23% of all GEPs.

**Table 3:** Expression of genes implicated in glycerol metabolism (Fig. 2) as determined from the three experimental datasets. Values of the gene expression change fold are given in Log2. Bold genes are considered to be over-expressed.

ORF	GENE	Fold Change in Gene expression																	
		Time after heat shock (min) in DB3								Time after heat shock (min) in DB2					Time after heat shock (min) in DB1				
				
		5	10	15	20	30	40	60	80	15	30	45	60	120	10	20	40	80	160
YIL155C	GUT2	1.95	2.27	2.06	-	3.54	2.24	1.76	1.08	2.49	1.95	1.22	1.81	1.21	-	-	-	-	-
YDL022W	GPD1	2.75	3.19	3.07	2.75	2.28	1.94	1.77	1.68	1.78	1.16	0.67	0.37	0.29	-	-	-	-	-
YOL059W	GPD2	0.26	0.41	0.49	-0.2	-0.1	0.19	-0.23	-0.23	-0.45	-0.57	-1.31	-0.16	-0.21	-	-	-	-	-
YML070W	DAK1	0.46	0.74	1.98	2.5	2.36	2.23	1.58	-	2.38	2.14	1.56	1.42	1.25	1.32	1.61	1.38	0.81	-0.12
YFL053W	DAK2	1.23	-	2.05	1.23	-	-	-0.18	-	-	0.9	-	-	0.89	1.44	0.21	0.72	0.19	0.37
YIL053W	GPP1	0.14	-0.06	-0.11	-0.09	-0.54	-0.12	-0.13	-0.09	0.13	0.51	0.5	0.65	-0.08	-	-	-	-	-
YER062C	GPP2	1.4	2.31	2.65	2.06	2.18	-	1.28	1.01	2.96	2.98	2	2.67	2.03	-	-	-	-	-
YGL084C	GUP1	-0.14	-0.09	0.76	0.7	0.52	0.62	0.12	0.12	0.59	0.76	0.77	-0.16	-0.36	-	-	-	-	-
YPL189W	GUP2	-	-	-1.84	-	-1.6	-	-0.58	-	-	-	-	-	-	-	-	-	-	-
YHL032C	GUT1	0.46	0.4	1.2	1.11	0.92	0.92	-0.17	-0.07	0.88	0.49	0.7	1.46	0.65	-	-	-	-	-
YLL043W	FPS1	0.03	0.06	0.2	0.19	-0.38	-0.1	0	-0.07	0.8	1.24	1.26	0.83	0.75	0.7	0.04	0.24	0.24	0.5
YDR368W	YPR1	0.5	0.71	1.01	0.65	-	0.24	0.39	0.38	1.37	1.39	1.16	0.42	0.7	-	-	-	-	-
YOR120W	GCY1	0.89	2.22	3.41	3.5	3.23	2.28	1.12	0.97	-	-	-	-	-	-	-	-	-	-

**Table 4:** Comparison of expression levels among the three data bases considered in this study.

Values for the Criteria from Experimental Dataset ^d^							
		Basal	DB1 10' ^a^	DB2 15' ^b^	DB3 10' ^c^	DB3 15' ^c^	DB3 20' ^c^
		
**C1**		60.2	263.8	222.4	175.0	262.6	199.4
**C2**		0.0012	0.0535	0.0343	0.1202	0.1830	0.1213
**C3**		1.8	7.7	3.7	4.9	10.3	7.7

**C4**	GLC	0.0345	0.0258	0.0184	0.0129	0.0102	0.0116
	G6P	1.01	16.13	11.83	18.88	18.08	10.73
	F16P	9.14	12.20	19.19	8.95	10.48	14.79
	PEP	0.0095	0.0065	0.0044	0.0041	0.0091	0.0051
	ATP	1.13	5.22	4.52	3.39	4.98	3.80

**C5**		0	9.52	7.92	9.87	11.78	10.29
**C6**		1.772	0.416	0.3902	0.4559	0.5146	0.4779
**C7**		71.42	9.41	11.81	4.19	7.79	11.41
**C8**		9.14	12.20	19.19	8.95	10.48	14.79

a: BD1 10': data from Eisen's experiments at 10 min. b: BD2 15': data from Causton's experiments at 15'. c: DB3 10', DB3 15' and DB3 20': data from Gasch's experiments at 10', 15' and 20', respectively. d: Units are the same as in Table 2.

**Table 5:** Quantiles of fold changes though all time courses. Values obtained from the pre-stress situation, minute 0, are characterized as noise.

Experimental Dataset	Minute	Quantiles				
		0.01	0.05	0.5	0.95	0.99
		
DB1	0	0.59	0.68	0.93	1.43	2.17
	10	0.17	0.34	0.89	2.35	7.78
	20	0.16	0.27	0.92	1.83	4.53
	40	0.24	0.37	0.90	1.77	2.93
	80	0.40	0.51	0.90	1.53	2.14
	160	0.36	0.48	0.97	1.88	2.91

DB2	0	0.43	0.57	1.00	1.42	1.57
	15	0.30	0.49	1.31	3.61	8.06
	30	0.51	0.76	1.62	3.86	6.92
	45	0.53	0.78	1.64	3.53	5.31
	60	0.44	0.66	1.46	3.34	5.39
	120	0.48	0.73	1.49	3.29	5.58

DB3	0	0.18	0.44	1.02	1.95	2.45
	5	0.24	0.44	0.89	2.30	8.06
	10	0.08	0.20	0.83	3.10	10.78
	15	0.05	0.19	0.93	4.29	16.22
	20	0.07	0.20	0.93	3.97	13.83
	30	0.11	0.24	0.92	3.73	12.21
	40	0.21	0.33	0.92	2.53	6.11
	60	0.40	0.54	0.97	1.78	3.25
	80	0.39	0.55	0.97	1.79	3.20
